# Understanding fertilizer adoption and effectiveness on maize in Zambia

**DOI:** 10.1016/j.foodpol.2019.05.004

**Published:** 2019-07

**Authors:** William J. Burke, Emmanuel Frossard, Stephen Kabwe, Thom S. Jayne

**Affiliations:** a Agricultural and Food Policy Consulting, Baltimore, MD, USA; b Group of Plant Nutrition, Institute of Agricultural Sciences, ETH Zurich, Switzerland; c Indaba Agricultural Policy Research Institute, Lusaka, Zambia; d Department of Agricultural Food and Resource Economics, Michigan State University, East Lansing, MI, USA

**Keywords:** Sub-Saharan Africa, Zambia, Agricultural productivity, Crop response, Fertilizer profitability, Soil quality

## Abstract

Increased fertilizer use will likely be crucial for raising and sustaining farm productivity in Africa, but adoption may be limited by ineffectiveness under certain conditions. This article quantifies the impacts of soil characteristics on maize response to fertilizer in Zambia using a nationally representative sample of 1453 fields, combining economic, farm management and soil analysis data. Depending on soil regimes, average maize yield response estimates range from insignificant (0) to 7 maize kg per fertilizer kg. For the majority of farmers, the estimated average value cost ratio is between 1 and 2, meaning fertilizer use would be fiscally rational, barring uncertainty and transfer costs. Since transfer costs exist and outcomes are uncertain, however, many farmers may sensibly pause before deciding whether to adopt fertilizer. This suggests shifting the emphasis of chronically low fertilizer use in Africa away from explanations of “market failure” toward greater emphasis on improving fertilizer efficacy.

## Highlights

• We combine data from economic and farm management surveys with soil sample analysis.
• We quantify the impacts of soil on response to fertilizer with 1453 sample fields.
• Critical thresholds are examined for pH, soil organic matter, soil texture and CEC.
• Maize yield response estimates range from nil to 7 maize kg per fertilizer kg.
• We consider profitability with respect to prices, risk aversion and transfer costs.

## 1 Introduction

Fertilizer use will be crucial for raising and sustaining farm productivity in Africa (Jayne and Rashid, 2013). Sustainable production requires that at least the nutrients removed through exportation of agricultural products be returned to the soil, but nutrient imbalance in agricultural production is currently one of the main threats to soil in sub-Saharan Africa (Jones et al., 2013, Montanarella et al., 2016). However, sustainably increasing productivity, or even just increasing productivity, is not always as simple as adding fertilizer. Soil characteristics are a key factor affecting crop response to fertilizer, and hence the profitability of and demand for fertilizer (Burke et al., 2017, Marenya and Barrett, 2009a, Matsumoto and Yamano, 2013). Specifically, the physical, biological and chemical characteristics of soils strongly influence the amounts of nutrients that can be stored and released, water availability, and the ability of plants to take-up nutrients (Jones et al., 2013).

The objective of this article is to quantify the impacts of soil characteristics on yields and yield response to fertilizer looking at the case of maize in Zambia.^1^ The importance of the relationship between soil characteristics and fertilizer effectiveness has received little attention in the literature on poverty, particularly in farmer survey-based studies, and in fertilizer promotion policies. A common and valid criticism of previous survey-based studies of yield response to fertilizers is that estimates are biased when there is correlation between fertilizer applications and omitted or unobserved variables. The typical argument states if fertilizer is more likely to be used on land where yields are more responsive, the estimated effect will be upwardly biased if soil quality is unobserved. Farmer ability is another commonly cited source of bias – the argument being positive correlation between skill and fertilizer application could make fertilizers seem more productive. This analysis is far less vulnerable to such omitted variable bias because we include specific soil quality indicators in the model, controlling for their effects (both direct and interacted with fertilizer application), thereby removing the omitted variable issues that plague other studies. We also control for farm management (timing of planting, timing of fertilizer application, seed and fertilizer application rates, seed type, and tillage methods) explicitly, and use proxies to further control for unobservable farmer characteristics.

We are able to control for these many possible sources of bias because of a uniquely comprehensive dataset. In 2012 the Indaba Agricultural Policy Research Institute surveyed a nationally representative sample of maize fields cultivated by 1653 rural households. In addition to an economic survey, farmers were asked details of farm management practices and field sizes were measured using global positioning system (GPS) trackers. From each field a composite sample of the first 20 cm of the soil profile were also collected and analyzed for several characteristics by the Zambia Agricultural Research Institute (ZARI). To the best of our knowledge, no data with this combined scope of geography and content has ever been studied.

We believe this article makes several contributions. First, we add to the few cases that use farm data to quantify soil-conditional yield response to fertilizer in Africa. Second, ours is a more rigorous examination of the relationship between soil and fertilizer than previous farm-based studies, because we incorporate multiple soil characteristics. The most thorough similar study to date is arguably Marenya and Barrett (2009b), who focus on the effects of soil organic matter (SOM) in Western Kenya. By contrast, we examine a broader range of important soil characteristics, including SOM, pH, cation exchange capacity (CEC) and texture. These characteristics are highly correlated, so it is important to emphasize the contribution of examining multiple characteristics. For one, the insights into the relationship between soil and productivity is more nuanced than is acknowledged by discussions focusing on a single soil characteristic. Also, while it is true that soil characteristics are correlated, they are not so highly correlated that different measurements cannot be simultaneously informative. For example, Marenya and Barrett (2009b) show that yields are more responsive to fertilizer on high SOM regimes, while Burke et al. (2017) show similar advantages on mid-range (neutral) soil pH. We will show that many soils could be classified as neutral pH and low SOM or high SOM and low (acidic) pH. Other imperfectly correlated soil characteristics further add to our nuanced soil typology. Most importantly, if farm management is aimed at affecting the important soil characteristics, looking at a wider scope of characteristics can illuminate a wider variety of possible interventions. As a minor additional contribution, we offer a brief discussion the different kinds of “soil data”, and how they may be more (or less) useful in combination with household survey data.

Finally, our analysis is more geographically expansive than prior efforts, based on a nationally representative sample of farm households in Zambia. It is worth highlighting that our respondents were chosen at random, meaning we estimate realized response rates, rather than the potential responses found using field trials or lead farmers. While estimates of potential yield response are often referenced in policy analyses, such studies tend to overestimate the response rates farmers can reasonably expect to achieve for many reasons (Snapp et al., 2014). We believe our representative approach more accurately describes crop response rates smallholder farmers actually obtain, and are thus more appropriate for evaluating policies.

The economic significance of the study is that it shifts the emphasis of chronically low fertilizer use in Africa away from explanations of “market failure”, high transfer costs and inadequate purchasing power, as important as these are, toward greater emphasis on highly variable and generally low profitability of fertilizer use because of response rates. Efforts to sustainably raise fertilizer use may depend on farmers’ ability to adopt management practices that favorably alter soil conditions. Policy makers can use the results of this study when designing comprehensive strategies to increase productivity while maintaining soil fertility.

Increased fertilizer use in Africa will likely be crucial to enabling the region to achieve rapid agricultural productivity growth. Most current efforts to promote fertilizer use focus on input subsidies (Jayne et al., 2018). If the additional revenue from fertilizer cannot cover the full economic cost of delivering it to farmers, however, fertilizer subsidy programs may be an unsuitable tool for promoting fertilizer use in the long run. The results of our study suggest more efforts in the areas of extension and adaptive research could be more beneficial.

The next two sections describe the conceptual and empirical frameworks for this study, followed by sections on data and estimation methods. The penultimate section presents results followed by our conclusions.

## 2 Conceptual framework: Soil data and the determinants of plant growth

This section discusses the nutrients plants need to grow, selected soil properties that determine nutrient storage and availability and other determinants of plant growth and yield response to fertilizers. Broadly speaking, the measurable soil-based determinants of plant growth, or “soil data”, can be categorized as either available *nutrients* or *general characteristics*.

### 2.1 Nutrients

Plant growth requires 17 nutrients, 14 of which are taken up from the soil solution. These are often sub-categorized as either micronutrients or macronutrients depending on the nutrient concentration in the plant.^2^ The macronutrients hydrogen, carbon and oxygen come from air and water and makeup the bulk of plant biomass. Nitrogen (N), phosphorus (P) and potassium (K) are taken up from the soil and are the most likely deficiencies on low productivity soils. Thus, unsurprisingly, these elements are the primary ingredients in many fertilizers used throughout the world.^3^ Besides the presence of nutrients, their relative importance to each plant also determines yield. Plants need nutrients in certain proportions; yield can be limited by nutrient imbalance if one is taken up in excess or in too small amount.

This rule for plant growth, popularly called the “law of the minimum”, was first posited by Carl Sprengel (1837) and later credited to Justus von Liebig (1840). A popular analogy is “Liebig’s Barrel”, where the amount of water held by a wooden barrel represents realized yield. Each plank of the barrel represents a required nutrient, and the length of the plank the availability of the same. Obviously, whichever plank is shortest determines the amount of water the barrel can hold.

The levels of available nutrients are the direct determinants of plant growth, so it would seem that measuring nutrients is the obvious entry point for using soil data to understand yield. However, we believe using nutrient data is not ideal when carrying out large-scale survey analysis for a few reasons. First, the timing of nutrient availability (and therefore soil collection) relative to a plant’s growth stage is very important, particularly for nitrogen. N is a very mobile element and does not stay in the soil solution long before either being taken up by a plant, washing away, escaping as a gas or leaching deep into the soil (Jones et al., 2013). Since the relevant timing is going to be specific to the plant, it is effectively impossible for any broadly representative household survey to measure relevant nitrogen consistently across observations.

Rather than measuring available nutrients directly, a more reliable practice is to measure the general soil characteristics that partially determine, and are highly correlated with the availability of nutrients.

### 2.2 Soil characteristics and nutrient availability

General characteristics can be sub-categorized as indicators of soil chemistry, soil physics or soil biology, and there are numerous ways to measure each. For a detailed scientific discussion we refer readers to Jones et al. (2013), but in this section we briefly discuss the characteristics used for this article and how they relate to productivity and expected yield response to fertilizers.

Soil chemistry can be quantified using cation exchange capacity (CEC), which indicates the soil’s capability to hold nutrients^4^ whether they come from inorganic fertilizer application or any other source. Sufficient for this discussion is to know that soils with higher CEC have a greater capacity to store nutrients, and are therefore likely to be more fertile (Jones et al., 2013). A second measure of soil chemistry is pH (potential hydrogen), which measures soil acidity. pH values range from 0 to 14, with 7 being neutral and lower (higher) values being more acidic (alkaline). Low pH hinders the retention of K (and other essential elements) while other nutrients like P can be strongly sorbed in acidic soils, limiting their availability even where they are found in the soil or applied as fertilizer. Soil microbial activities and root growth are also negatively influenced at low pH values. In short, low pH soils tend to be less productive (Jones et al., 2013).

A common soil biology measure is soil organic matter (SOM).^5^ Marenya and Barrett, 2009a, Marenya and Barrett, 2009b and subsequent agricultural economics literature refer to SOM as “the” best measure of soil quality. Indeed SOM is an indicator for several important factors; higher SOM suggests higher levels of nutrient stocks, higher capacity to hold water, and suggests soil structure that ensures good growth conditions for roots. All together, higher SOM is expected to be associated with higher yields and higher yield response to fertilizer (as found in Kenya by Marenya and Barrett (2009b) and Uganda and Kenya by Matsumoto and Yamano (2013)).

**Fig. 1:**
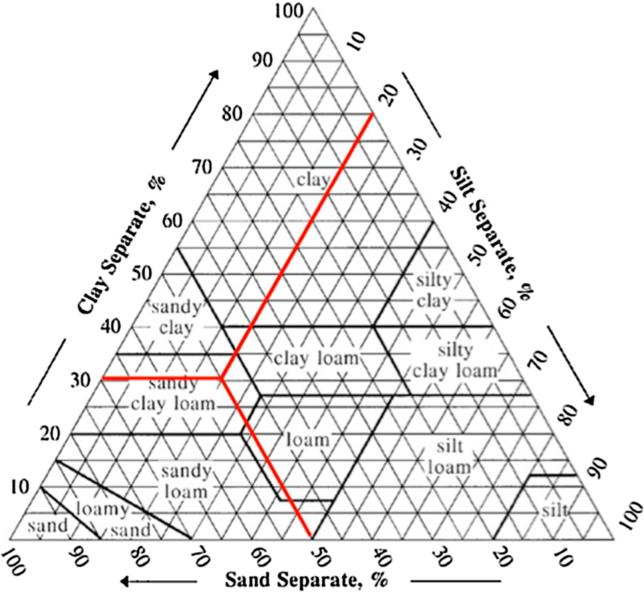
USDA soil texture triangle. For example, a soil that is 30% clay, 20% silt and 50% sand (shown by red lines) is “sandy clay loam”. Source: Adapted from USDA, online at: http://www.nrcs.usda.gov/wps/portal/nrcs/detail/soils/survey/?cid=nrcs142p2_054167.

We measure soil physics using texture classification, or the tangible makeup of the soil. All soil is made up of particulates classified as either clay, silt or sand according to its size (with clay being the smallest and sand the largest). We use the United States Department of Agriculture (USDA) soil texture classifications as identified by the “texture triangle” (Fig. 1). While texture classification may seem to rely on tediously precise measurements, it is actually one of the simplest and most dependable characteristic tests available; a properly trained person can classify soils with a high degree of accuracy without the use of laboratory equipment (Thien, 1979). Generally speaking, higher clay content soils tend to be more productive because they can hold moisture and nutrients longer than coarser soils.

### 2.3 Fertilizer and other determinants of yield response

In addition to soil characteristics, we model yield as a function of management practices and other control variables. First, Zambian farmers apply either “basal” fertilizer, which is 10% nitrogen, 20% phosphates and 10% potash (potassium) or NPK = 10-20-10, or “top dress” fertilizer, most commonly urea (NPK = 46-0-0). Basal is meant to be applied at the time of planting or earlier, while top dressing is best applied after plants have germinated and begun to grow. Of course, any fertilizer could be applied, but virtually all fertilizer used on maize in Zambia (>99%) is one of these two.

As suggested, the timing of fertilizer application is a factor that affects yield response on farmer-managed fields. Phosphorus availability is especially important at early stages of maize growth. Used as a building block of DNA, RNA, ATP (for photosynthesis) and phospholipids, phosphorus regulates cell division during early growth, and is key for the development of roots and protein formation (Jones et al., 2013). Late basal application can thus be expected to have a negative effect on yield response.

### 2.4 Other control variables

Other control variables include the plant variety (sowing “improved” hybrid and OPV seeds or not), seed application rate (kg/ha), and the timing of planting with respect to the beginning of the rainy season. Planting before or near the beginning of the first rains is beneficial since plants can then take advantage of moisture and the annual “nutrient flush” (the release of nutrients from organic material that has decomposed since the previous rainy season) (Haggblade et al., 2011). According to ZARI (2002), maize is typically considered planted “on time” if it is planted in November to early December. Finally, a series of indicator variables control for yield differences related to tillage methods (ripping, ridging, bunding, plowing, basin, or zero tillage, or traditional hand-hoeing).

A wide range of additional data were collected regarding field management, including rates for liming, irrigation, agroforestry tree use, crop mixing, insecticide, herbicide, manure and compost, flooding and flood prevention. However, there are too few observations in our data to identify the effects of employing these techniques (from 5% to less than 1% of households employed each of these practices). We also cannot control for the effects of weeding due to lack of variation, but for the opposite reason – 99% of the fields in the sample were weeded.^6^

We control for unobserved factors related to farmer ability and labor availability using proxy variables. Specifically, we include the education of the household head, the family land to labor ratio and an indicator for whether the family hired labor. While these variables fit the definition of good proxies (i.e., are likely correlated with unobserved ability), they are not as detailed as would be ideal (e.g., we cannot provide more precise information on the amount of hired labor). It is therefore best to regard proxies as control variables and their coefficients should be interpreted with caution.

## 3 Empirical model of maize yield and response to fertilizer

Following the above framework, the explanatory variables in the empirical model for *yield* (kg/ha)^7^ include: the number of weeks after planting for basal application (*weeks*), the seeding rate (*seedrate*, kg/ha), a binary variable equal to one if the seed used was a hybrid or open pollinated variety (*hybrid*) instead of local, a binary variable equal to one if planting occurred before the ZARI recommended timeframe (*early*) and another indicator if planting occurred after the ZARI recommended timeframe (*late*), with the effect of being “on time”, or from pre-November through the second week of December (ZARI, 2002) being subsumed into the intercept. We also include a series binary variables for tillage methods (***tillage***), including the use of planting basins, zero till, plowing, ripping, ridging, or bunds. The effect of traditional hand hoeing is subsumed into the intercept. Control variables that act as proxies for farmer ability and labor availability are years of education for the household head (*educ*), the land to labor ratio measured as the number of hectares the household cultivates divided by the number of household members over 12 years of age (*landlabratio*), and a binary variable equal to one if the household hired additional labor (*hirelab*).

Key variables used to measure yield response are fertilizer (*fert = basal + topdress*, all measured as kg/ha), application timing (*weeks*), and soil characteristics. We will be using more than one soil characteristic within a single model, but for the time being we give these general names *soil*1*reg* and *soil*2*reg*, which are binary indicator variables for the regime (low = 0; high = 1) of the first and second soil characteristics included in the model. For example, if the first soil characteristic is SOM and the second is pH, a soil that is “high” SOM but “low” pH will have *soil*1*reg = 1* and *soil*2*reg* = 0.

The entire yield function is modeled as quasi-linear conditional on one of these soil characteristics (*soilchar2*), as in Marenya and Barrett (2009b). To incorporate additional soil information, yield response is also conditional on one other soil characteristic (*soilchar1*) within each linear component of the model. The process for defining “high” and “low” regimes will be described in the next section, but to avoid overstating our model’s complexity, we would like to make it clear that “quasi-linear” means the model within each *soilchar2* regime will take a linear functional form, so that, for example in the “low” regime for *soilchar2*:

(1) $$E\left(yield|soil2reg=0\right)=\alpha_{0}+\beta_{0,1}fert+\beta_{0,2}{\mathrm{fert}}^{2}+\beta_{0,3}soil1reg+\beta_{0,4}soil1reg*fert+\beta_{0,5}basal*weeks+\beta_{0,6}seedrate+\beta_{0,7}{\mathrm{seedrate}}^{2}+\beta_{0,8}hybrid+\beta_{0,9}early+\beta_{0,10}late+\beta_{0,11}tillage+\beta_{0,12}educ+\beta_{0,13}landlabratio+\beta_{0,14}hirelab$$

The first number in the subscript for each parameter corresponds to the value of *soilchar2*, so each parameter described in Eq. (1) has a corresponding analogue under the “high” *soilchar2* regime (e.g., $\beta_{1,1}$ will be the coefficient on *fert* when *soil*2*reg* = 1).

Since we estimate unique yield responses to fertilizer for each observation, much of our interpretation of results focuses on the expected average products (APs) of fertilizer. The AP is the additional kilograms of maize attributable to fertilizer use, divided by the kilograms of fertilizer applied. Using Eq. (1) again as an example, for basal fertilizer on the “low” regime of *soilchar2,* the expected AP reduces to:

(2) $${E\left({\mathrm{AP}}_{\mathrm{basal}}|soil2reg=0\right)=\beta }_{0,1}+\beta_{0,2}\left(basal+2topdress\right)+\beta_{0,4}soil1reg+\beta_{0,5}weeks$$

And a similar equation could be shown for E(*AP_basal_|soil*2*reg* = 1) as a function of observed values of *basal*, *topdress*, *soil*1*reg*, *weeks*, and the parameters $\beta_{1,1},\beta_{1,2},\beta_{1,4},\;and\;\beta_{1,5}$. Likewise, it is straightforward to derive the function for expected APs of *topdress*.

For any observation, the farmer-specific AP can be computed as a function of parameter estimates and observed data. With these calculations in hand, the mean of the AP can be calculated according to soil characteristic groups (or any other categorization). For the summary statistics discussed in our results section, the aggregation of APs for each fertilizer type will only include those who actually applied that fertilizer type. We will also discuss cumulative distributions of the APs across the country.

## 4 Data

**Fig. 2:**
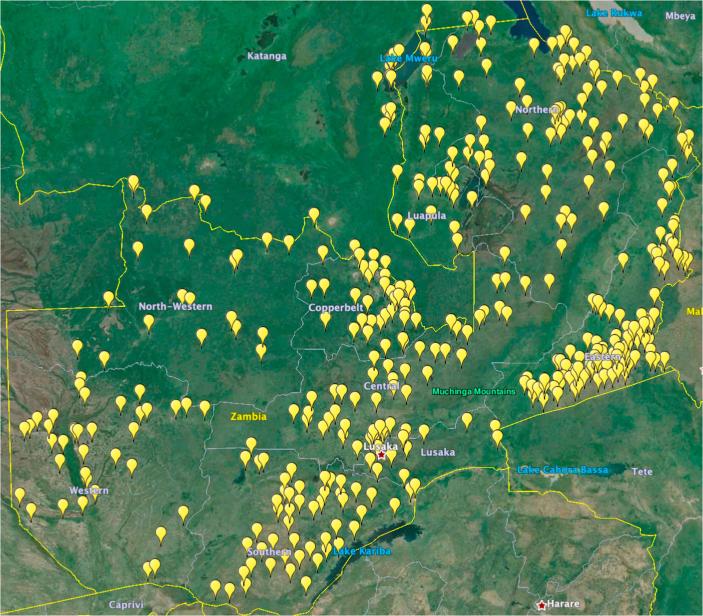
Village locations where soil samples were collected (4 samples each). Source: RALS12, Google Earth.

The sample for this survey is a sub-set of the households interviewed during the Rural Agricultural Livelihoods Survey (RALS) carried out in May and June 2012 as a collaborative effort between the Zambian Ministry of Agriculture and Livestock (MAL), the Central Statistics Office (CSO) and the Indaba Agricultural Policy Research Institute (IAPRI). Standard enumeration areas (SEAs), designated by CSO for census purposes, were selected using probability proportional to size, and a constant sample size of 20 households was surveyed in each SEA. The RALS survey instrument covered a broad range of household economic data covering the 2011 harvest and 2012 marketing season.^8^ Within the 20 selected observations in each SEA, 4 were chosen at random to be included in the sub-sample used for this analysis. In addition to collecting soil samples, another small survey collected information related to the 2012 harvest for the largest maize field from the sub-sample.^9^ The area of each maize field in our sample was measured using a GPS device. For a full description of sampling methods see IAPRI (2012). The sample village locations are illustrated in Fig. 2.

We chose the largest maize fields because we wanted to estimate the yield response on the fields most important to total production, and it simplified standard protocol. We acknowledge the caveat, however, that using only the largest field might introduce some sampling bias (e.g., if the largest maize fields tend to be more or less productive than other maize fields). It is thus worth noting that the majority of households in the RALS (over 60%) have only one maize field to choose from. However, if our mean effects are drastically different than those found in earlier studies, this possible sampling drawback should be considered.

1714 soil samples and largest maize field (LMF) surveys were collected from 1680 households. Soil samples outnumber households because fields with noticeable differences in slope or soil (e.g. color or texture) were sampled more than once (26 households provided 2 samples each and 4 households provided 3 samples). Data are aggregated to the field level as un-weighted means across soil samples. Of these 1680 households, 12 are excluded because they lack corresponding farm management data due to either enumerator or data entry errors that are treated as random occurrences.

Of the 1668 remaining, 2 observations used fertilizer without reporting quantities and are thus dropped from econometric analysis. There are 10 replacement households in the LMF survey sample that were not included in the RALS sample because respondents were unavailable.

Twenty-three observations experienced total crop loss and harvested no area, thus having undefined yields. Of the remaining (N = 1633), 157 fields were in a wetland area, and are excluded from our analysis. A Chow test confirms the structural difference in the relationships between production factors and yields for this group and the remainder of the sample. Finally, 23 of the remaining observations report harvests on small fields that would correspond to yields greater than 14.5 mt/ha (three standard deviations above the mean), which are excluded as outliers. As such, the final data we work with is from 1453 field-level observations.

Enumerators and their supervisors were trained by ZARI to collected soil samples. Each sample analyzed was a composite of 10–20 sub-samples of soil collected throughout each field. The protocol for the number of sub-samples and the collection pattern throughout the field followed by enumerators was specified according to field size. Each sub-sample was a composite of equal parts soil in the 0–10 cm and 10–20 cm depth horizons, where maize root density is highest. For fields planted using ridge tillage, samples were taken directly from the ridges. A full description of the methods used to collect samples is described in IAPRI (2012).

Soil texture was assessed manually in the ZARI laboratory following the methods described by Thien, 1979, Presley and Thien, 2008. The manual estimation of texture is fairly standard globally and is known to give reasonably accurate results. Although more rigorous approaches exist, they require material which was not available at ZARI. Soil pH was analyzed in 0.01 M CaCl_2_ (calcium chloride) solution, which is also consistent with global standards. Soil organic matter was measured following the Walkley and Black method (Walkley and Black, 1934). Again, this procedure is commonly used, especially in many labs in developing countries. More precise measurements might be possible if a precision balance, gas chromatograph, and the necessary consumables were available, but at ZARI that was not possible. CEC was analyzed using the ammonium acetate method at pH 7.0 and measurement of the sorbed ammonium (NH_4_) by titration following the exchange of sorbed NH_4_ with excess sodium chloride (NaCl). Measuring CEC at the fixed pH of 7.0 is a procedure developed for soils with permanent charges, such as those found in Europe, but is less reliable for soils with variable charges (e.g., rich in Fe and Al oxides and in 1:1 clay as kaolinite –which as a low shrink/swell capacity – but low in 2:1 clay as smectites –which swells more when wet–), such as those found in Zambia. In these soils, it is generally considered more appropriate to measure the “effective CEC” at soil pH. In the 50s and 60s, however, the tradition was to measure the CEC at pH 7.0 as the importance of variable charges had not been realized, and that method is still regularly used by scientists working in Africa. The ZARI laboratory operators were trained by a team of soil scientists from the University of Wageningen and follow protocols that can be implemented with the locally available equipment.

Employing ZARI’s laboratory for soil analysis has obvious advantages, not least of which are their presence in Zambia, their willingness to train soil sampling teams and a collegial relationship with the policy makers that stand to benefit from this study. One important drawback is that the ZARI laboratory is not formally accredited, meaning no recent, independently evaluated data are available to determine the reliability of our results. Specifically, it is useful to scrutinize the accuracy and precision (or respectively the external and internal validity) of test results. Accuracy, or the closeness of test results to actual values, is typically evaluated by comparing blind test results to known parameters from fabricated samples. Unfortunately this was not possible for our study (and should be a priority moving forward).

Precision refers to the repeatability of test results – do the same tests on the same samples produce similar results? Less variation within multiple tests of the same sample indicates greater precision. While we cannot demonstrate the accuracy of the ZARI results, confidence in the lab’s precision validates comparing soil characteristics across observations in our sample. For example, a pH result measured as 7 may or may not be truly neutral, but if the lab has a high measureable degree of precision, we can be confident that the sample is less acidic than another sample with lower pH results from the same lab.

**Fig. 3:**
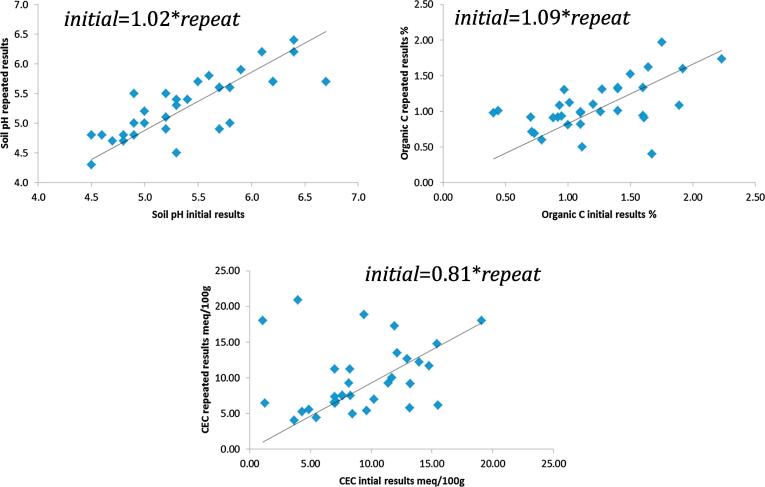
Assessment of the precision of ZARI laboratory results. Sources: RALS12 soil samples, ZARI analysis and authors’ calculations.

To evaluate ZARI’s precision, 2% of our observations were randomly selected for a second round of testing and comparison to initial measurements. Precision-check results are illustrated in Fig. 3. Clockwise, starting from the top left, each panel is a scatter plot of initial results on the horizontal axis and re-testing results on the vertical axis for pH, organic carbon and CEC. Observations on the 45° line (shown) are those with the exact same result in both tests.

To formalize the comparison we report results from regressing the initial results on the re-test results without an intercept (thus, a coefficient estimate of “1” would suggest precise measurement). In each case our estimate is substantively close to 1 and we fail to reject the hypothesis that each coefficient is one (p < 0.05). The R-squared comparing initial to re-test results for pH, organic carbon and CEC are 0.996, 0.91 and 0.75 respectively.

Texture is a categorization rather than a continuous variable, and so it doesn’t lend itself to this type of precision evaluation. That said, this is one of the simplest tests to carry out, and such analyses are seldom questioned. Also, we include texture in our model as more general categories according to clay content (see below), so the effects of potential measurement error are likely to be low.

Soil analysis and other explanatory variables are summarized in Table 1. According to ZARI, the large variability observed for organic C, CEC and soil pH is credible. Variability in CEC and soil pH, for example, could be driven by strong differences in soil weathering observed across the country which includes strongly weathered soils (e.g., ferralsols and podzols), soils that are little weathered (cambisols and luvisols) and saline soils (Mambo and Phiri, 2003). Similarly, high organic matter could be explained by excessive soil moisture as in histosols.

**Table 1:** Summary of Zambian field-level soil analysis and survey data.

*Variables*	Mean	St. dev.	N
Yield (maize kg/ha)	2256	2016.2	1453

*Soil chemistry/biology*			
Soil pH (Acidity)	5.4	0.61	1453
Organic Matter (%)	1.8	0.70	1453
Cation exchange (meq)	9.5	5.42	1453

*Texture groups^a^ (1 = yes)*			
1 (Less than 5% clay)	0.18	0.38	1453
2 (5–25% clay)	0.26	0.44	1453
3 (25–40% clay)	0.50	0.50	1453
4 (More than 40% clay)	0.07	0.25	1453

*Fertilizer*			
Basal application rate (kg/ha)	177	137.2	957
Top dress application rate (kg/ha)	170	133.4	980
Weeks after planting before basal applications	3.4	1.6	957

*Tillage methods (1 = yes)*			
Hand hoe	0.29	0.45	1453
Planting basins	0.02	0.15	1453
Zero tillage	0.01	0.09	1453
Plow	0.36	0.48	1453
Ripping	0.01	0.10	1453
Ridges	0.28	0.45	1453
Bunds	0.03	0.17	1453

*Other management*			
Seed application rate (kg/ha)	36.1	47.6	1453
Hybrid/OPV seed use(1 = yes)	0.61	0.49	1453
Early planting^b^ (1 = yes)	0.04	0.20	1453
Late planting^c^ (1 = yes)	0.23	0.42	1453

*Proxies*			
Education of hh head (years)	6.4	3.96	1453
Land-labor ratio^d^	0.58	0.57	1453
Hired labor (1 = yes)	0.17	0.37	1453

Source: RALS 2012 and ZARI soil analysis.

Notes: For binary variables (where 1 = yes), the mean is the share of observations. a – Texture categorizations are detailed in the main text and are roughly up to 5% clay (group 1), 5–25% clay (group 2), 25–40% clay (group 3) and over 40% clay (group 4). b – Early planting is finished before November. c – Late planting is finished after the second week in December. d – The land to labor ratio is cultivated hectares divided by the number of household members over 12 years of age.

Overall we conclude the sum of evidence suggests ZARI test results have an acceptable level of precision for the present analysis, but we are not able to evaluate the laboratory’s accuracy. The implications of these conclusions vis-à-vis our estimation approach is discussed in the next section.

## 5 Estimating yield response to fertilizer

Soil characteristics enter our model’s yield function as interaction terms and by specifying a quasi-linear functional form. The effects of fertilizer and other determinants are allowed to differ systemically depending on whether soil characteristic measurements are above or below estimated threshold levels. The concept of thresholds in the process of plant growth is well established in the agronomic literature; a nice illustration of this is the “law of the minimum” mentioned earlier.

Marenya and Barrett, 2009a, Marenya and Barrett, 2009b use this principal to motivate a quasi-linear yield model using data from Western Kenya and soil organic matter as the threshold variable. Matsumoto and Yamano (2013) used a similar model and procedure to study fertilizer effects in parts of Uganda and Kenya. Burke et al. (2017) apply the threshold principle to yield response to fertilizer with respect to soil pH levels. In the latter case, however, thresholds were imposed *a priori* (not estimated).

### 5.1 Model specification with respect to soil characteristics

Our extension of previous threshold models is that we do not rely on just one measure of soil quality. Specifically, we use data for four different measures (pH, CEC, SOM and texture). It is infeasible to use all of these in the same model because we do not have sufficient data. Allowing for soil regimes requires sample splitting, and our sample size becomes too small if we split along several soil thresholds simultaneously. Instead, the two types of thresholds we have proposed (one interacting with only fertilizer, a “type 1” regime-defining variable, and the other structurally shifting the whole production regime, a “type 2” regime-defining variable) allow us to consider up to two soil characteristics in each model.

For our primary analysis we discuss two models: 1) allowing yield and response to fertilizer to be conditional on pH and SOM, and 2) we replace SOM with soil texture. We have chosen to focus on pH and SOM because of the relative measurement precision compared to CEC (Fig. 3), and because ZARI procedures for measuring pH and SOM are more in line with global standards. We include texture in our primary focus because it is one of the most reliable characteristics to test. These three measures also cover all three types of soil characteristics (biology, physics and chemistry). That said, many alternative specifications are possible with our data, and the story emerging from the models on which we focus is consistently born out under other specifications. Results from several other models, including some using CEC, confirm the robustness of our findings. Though full results are not presented in the main paper, they are summarized in Fig. 5 and briefly discussed below.

Since texture is a categorical variable, we do not formally estimate thresholds. Rather, we group textures into four categories according to clay content. The first group of soils (sand, loamy sand and silt) have virtually no clay, those in group 2 (sandy loam, loam and silt loam) are roughly between 5 and 25% clay, group 3 (sandy clay loam, clay loam and silty clay loam) is about 25–40% clay, and group 4 (sandy clay, silty clay and clay) is 40–100% clay. These categories represent 18%, 26%, 50% and 7% of our observations respectively.^10^ For all other soil characteristics we estimate thresholds as described presently.

### 5.2 Estimating thresholds

Approximate values at which we might expect to find threshold effects can be found in the literature. For example, George, Horst, and Neumann (2012)^11^ suggest a critical value for pH is 5.5, which is higher than, but consistent with the ZARI maize production guidelines that place the critical value between 4.4 and 4.8 depending on soil type (ZARI, 2002). There is no official ZARI guideline for SOM, but interviews with heads of ZARI research and extension divisions indicated we might expect to find a SOM threshold between 1% and 2%. The ZARI maize production guidelines also indicate denser (less sandy) soils would likely be more productive, though no specific values are discussed, nor is CEC (ZARI, 2002). Despite some extant knowledge, however, we maintain it is best practice to estimate, rather than impose thresholds. Since our independent assessment of the lab cannot attest to the accuracy of our data but gives us confidence in its precision, we would rather rely on the evidence-supported internal validity of our data than the unknown degree of external validity.

To discuss how thresholds are estimated, recall we allow for two types of threshold effects (the aforementioned “type 1” and “type 2”). A type 1 threshold variable (*soilchar1*) acts to shift expected yield and it’s response to fertilizer (only) depending on whether *soilchar1* is above or below a threshold to be estimated, ${(\theta }_{1})$. Using a stylized version of equation (2), this can be written as:

(3) $$\mathrm{yield}=\beta_{1}fert+\beta_{4}soil1reg+\beta_{5}\left(fert\cdot soil1reg\right)+X\gamma$$

$$where\;soil1reg=\left\{\begin{array}{c} 0\;if\;soilchar1\leq \theta_{1}\\ 1\;if\;soilchar1>\theta_{1} \end{array}\right)$$

where** *X*** is the vector of other control variables previously described. β and γ are coefficients and $\theta_{1}$ is the threshold parameter, all to be estimated.

A type 2 regime-defining threshold variable (*soilchar2*) affects the entire production function depending on whether *soilchar2* is above or below a (different) threshold to be estimated ${(\theta }_{2})$, as in:

(4) $$\mathrm{yield}=\left\{\begin{array}{c} \beta_{11}fert+\beta_{21}soil1reg+\beta_{31}\left(fert\cdot soil1reg\right)+X\gamma_{1}\;if\;soilchar2\leq \theta_{2}\\ \beta_{12}fert+\beta_{22}soil1reg+\beta_{32}\left(fert\cdot soil1reg\right)+X\gamma_{2}\;if\;soilchar2>\theta_{2} \end{array}\right)$$

At this point, two questions emerge: (1) Why have two types of thresholds, and (2) how should they be estimated? The answer to the first question is a simple matter of balancing flexibility with feasibility. The most flexible way to include soil characteristics in the model would be to treat them all as type 2 regime-defining variables, but this would require more data than we have available. So, to allow for the possibility of having more than one measure of soil quality in a single model, we impose restrictions so that one measure affects only response to fertilizer and the intercept.

For continuous regime-defining variables, both of the threshold values $\theta_{1}$ and $\theta_{2}$ are estimated using a grid search approach similar to that used by Marenya and Barrett (2009a), which is also common in threshold cointegration literature (Balke and Fomby, 1997). Simply put, we estimate the model under all feasible assumptions regarding the values of $\theta_{1}$ and $\theta_{2}$ and our estimates are the thresholds that best fit the data.

Since we have two threshold values to estimate, an iterative process is employed. First we estimate ${\hat{\theta }}_{1}$ using an ordinary least squares (OLS)-based grid search and the full sample (assuming $\left(\theta_{2}=0\right)$). Second, we estimate ${\hat{\theta }}_{2}$ assuming $\theta_{1}={\hat{\theta }}_{1}$ is the same in both *soilchar2* regimes. Third, we update our prior, now assuming $\left(\theta_{2}={\hat{\theta }}_{2}\right)$ and re-estimate ${\hat{\theta }}_{1}$ within each *soilchar2*-based regime. Call these new estimates ${\hat{\theta }}_{11}$ and ${\hat{\theta }}_{12}$ for the low *soilchar2* and high *soilchar2* regimes respectively. A specified convergence rule could be applied and this process could go on until it is satisfied, but in each of our cases using the first estimates of ${\hat{\theta }}_{11}$ and ${\hat{\theta }}_{12}$ produces the same estimate of ${\hat{\theta }}_{2}$ as in the first iteration (so no further iterations are necessary).

To justify splitting the sample into two *soilchar2*-based regimes we conduct a Chow test. If we reject the null hypothesis that the yield function is no different between regimes, OLS within each regime (holding standard assumptions) provides unbiased estimates and the usual t-statistics can be used for inference. Since the estimates ${\hat{\theta }}_{2}$, ${\hat{\theta }}_{11}$ and ${\hat{\theta }}_{12}$ do not have a known distribution, we conduct hypothesis testing on them using bootstrapped standard errors, just as in Hansen, 1996, Marenya and Barrett, 2009a.

### 5.3 A comment on endogeneity bias from omitted variables

In the introduction we briefly discussed the frequent criticism of studies quantifying yield response to fertilizer using survey data due to the susceptibility to endogeneity bias or, more specifically, correlation between fertilizer use and unobserved factors. It is worth revisiting how this may affect our results and any caution that should be taken when considering them. A typical and valid argument is that if fertilizer is more likely to be used on land where yields are more responsive, the estimated effect will be upwardly biased if soil quality is unobserved. This study is far less vulnerable to such endogeneity problems than earlier efforts because we include specific soil quality conditions in the model, controlling for their effects (both directly and as interactions with fertilizer application) and thereby minimize the omitted variable/selection bias issues. These soil condition variables are arguably the best proxies for soil quality used in any analysis of farmer-based survey data to date. Including soil data is also superior to controlling for unobserved fixed effects using panel data or controlling for correlation with omitted variables via an instrument because, while those methods “control” for omitted variable effects in theory, our method allows us to actually estimate the impact of soil quality, and the effects soil quality has on fertilizer efficacy. In other words, we control for the important determinants of yield that might be correlated with fertilizer use in our model, as opposed to through our choice of estimator, which allows for a more nuanced understanding of fertilizer effects.^12^

The argument that our model is not severely burdened by endogeneity is bolstered by the fact that the average of yield responses we report across soil regimes in our results are in line with those from several other recent studies, many of which, while lacking nuanced model specifications, address endogeneity using various statistical approaches (Jayne et al., 2018).

Still, we can consider the implications of any remaining bias caused by unobserved soil quality information. Because soil quality tends to be positively correlated with both yields and fertilizer use, any remaining bias caused by missing information is likely to be positive. As will be shown, our estimates of yield response to fertilizer are quite low and a major conclusion of this study is that low crop response to fertilizer is a key reason fertilizer use by Zambian farmers is lower than some might expect. So, even if an omitted variable bias remains, the “true” crop response to fertilizer would likely be lower than our estimates, which would only strengthen the conclusions about low response rates to fertilizer. So, we believe our approach explicitly controls for soil quality, and our conclusions can be considered robust to any potentially remaining bias caused by omitted soil characteristics.^13^

## 6 Results

We report the results one model specification at a time as described in the sub-headings according to which soil characteristic is used as each threshold type.

### 6.1 Model 1: SOM = type 1 and pH = type 2

Select estimate results from the model including SOM and pH are shown in Table 2 (several controls are included in regressions as described, but results are not shown to conserve space. Full results are available). To estimate this model we first search for a SOM threshold affecting only yield and fertilizer response. This is initially identified at 1.4% (p = 0.00; 95% CI = [0.7, 2.1]). Then, assuming this threshold holds throughout the model, we search for a pH threshold to identify two regimes for the entire production function. The total sum of squared residuals for the model is minimized at the pH threshold level of 5.4. A Chow test rejects the hypothesis that these regimes are no different (p = 0.00) and the bootstrapped standard error suggests the threshold value itself is statistically significantly different from zero (p = 0.00; 95% CI = [5.0, 5.8]). Finally, within each pH regime, we re-evaluate the potential SOM thresholds and estimate little or no change at 1.2% and 1.4% for the low and high pH regimes respectively. Since these are well within the 95% confidence interval of our initial estimate, we do not continue the iterative threshold identification process. It is worth noting that pH and SOM thresholds are close to *ex ante* expectations.

**Table 2:** Select results for understanding maize yield and response to fertilizers in Zambia with SOM as type 1 and pH as type 2 threshold variables.

*Estimated threshold parameters*^a^		

Type 2-pH	5.4^***^	
	(0.18)	
Type 1-SOM on low pH regime	1.2%^***^	
	(0.42)	
Type 1-SOM on high pH regime	1.4%^***^	
	(0.25)	

*Select regression results*	pH ≤ 5.4	pH > 5.4

Fertilizer rate	3.647^***^	2.089^+^
	(0.72)	(1.30)
Fertilizer rate squared	−0.001^***^	0.001
	(0.00)	(0.00)
High SOM	−308.13^*^ (159.9)	−488.51^*^ (287.2)
High SOM * Fertilizer rate	2.157^***^	3.434^***^
	(0.61)	(0.86)
Weeks delay * Basal fertilizer rate	−0.278	−0.593^+^
	(0.26)	(0.36)

N	831	622
R^2^	0.51	0.44
Weighted mean R^2^	0.48	

Robust standard errors in parentheses. *, **, *** Indicate significance at the 10%, 5% and 1% levels respectively, + indicates significance at the 11% level. Chow test rejects structural equality between Type-2 (pH) regimes (p = 0.00) a- threshold standard errors are based on 50 bootstrapped replications.

To better understand these results, the mean APs (amongst fertilizer users) of the two types of fertilizer used in Zambia are presented in Table 3a, Table 3b by pH and SOM regimes. The first (3a) shows the AP for basal and the second (3b) shows the AP for top dressing. Again, the key difference between the two is that the AP of basal is a function of application delays while the AP for top dress is not.

**Table 3a:** Matrix of average product (kg/kg) of basal fertilizer for maize production in Zambia (Model 1).

		pH level	
		Low ≤ 5.4	High > 5.4
Soil organic matter	High	4.25^***^ (0.54) [n = 495]	4.00^***^ (0.82) [n = 294]
	*Threshold*	*1.2%*	*1.4%*
	Low	1.97^***^ (0.66) [n = 107]	0.46 (0.87) [n = 91]

Source: RALS 2012. Delta-method standard errors in parentheses, sub-sample sizes in brackets. Note – APs include fertilizer users only.

**Table 3b:** Matrix of average product (kg/kg) of top dress fertilizer for maize production in Zambia (Model 1).

		pH level	
		Low ≤ 5.4	High > 5.4
Soil organic matter	High	5.15^***^ (0.69) [n = 495]	5.81^***^ (0.78) [n = 294]
	*Threshold*	*1.2%*	*1.4%*
	Low	2.93^***^ (0.70) [n = 107]	2.38^**^ (1.13) [n = 91]

**Fig. 4:**
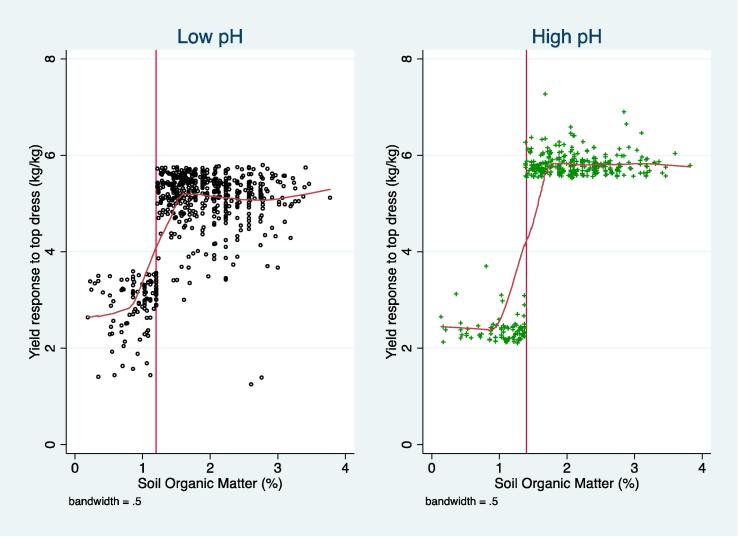
Scatter plots and LOWESS regression of average product of top dress by soil regimes for model 1: SOM = Type 1 threshold and pH = Type 2 threshold. Source: RALS 2012. Notes: Each dot indicates an individual observation’s estimated average product of fertilizer. Estimated pH thresholds are indicated by vertical lines. Limited to observations where SOM is less than 4.6% (3 observations not shown).

For both types of fertilizer, SOM regime effects are consistent with expectation and in line with earlier results reported in Marenya and Barrett (2009a) and elsewhere. Specifically, for a given type of fertilizer and pH regime, the mean AP is between 2.2 and 3.5 kg of maize per kg of fertilizer higher on soils with higher SOM levels. This amounts to a difference between 75% (top dressing on low pH soil) to over 800% (basal fertilizer on high pH soil). This is further illustrated on Fig. 4, which plots all estimated response rates for top dress across soil regimes.

For top dress, where SOM is above the critical threshold, the average product of fertilizer is greater on higher pH soils, as expected (5.8 kg/kg versus 5.2 kg/kg). On lower SOM soils, on the other hand, the estimated AP of top dress is slightly higher on acidic soils, which is unexpected, but the difference is small in absolute terms (0.45 kg/kg) and the *difference* not statistically significant (though each estimate is significantly different from zero individually). Similarly, the estimated AP for basal fertilizer is higher on more acidic soils regardless of SOM regimes (Table 3a, Table 3b), but this unexpected result can largely be accounted for by application delays. In short, the relationships between pH and SOM are not as direct and simple as one might expect, but our results are largely consistent with the agronomic underpinnings of the model – under similarly “good” conditions, more neutral pH and higher SOM lead to more beneficial maize yield response to fertilizers. The risk of low response rates is greatest on more acidic soils, which is consistent with known agronomic principles. Fig. 4 also shows that the highest APs (response rates) are found on soils that are in both high pH and high SOM regimes (in addition to previously cited material, we recommend Tully et al. (2015) for more insights on the complex relations between SOM and pH).

Table 2 also underscores the importance of controlling for delayed application when examining the APs of basal. On high pH soils, where yield response has the highest potential, for every week after planting basal fertilizer application is delayed on acidic soils, the expected AP decreases by about 0.6 kg/kg. The two-sided test shows this difference is significantly different from zero at the 11% level, while one-sided tests fail to reject the hypothesis that delays have a strictly negative effect and reject the hypothesis that the effect is non-negative (p = 0.06)

### 6.2 Model 2: Clay content = type 1 and pH = type 2

Table 4 shows select results from an alternative model where soil texture categories replace SOM as the type 1 threshold variable. In this model, soil texture categories are established prior to estimation and a grid search is employed to identify the pH regimes that minimize the sum of squared errors. Here the estimated pH threshold is found at 5.6 (p = 0.00; 95% CI = [5.0–6.2], which is again close to *ex ante* expectations.

**Table 4:** Select results for understanding maize yield and response to fertilizers in Zambia with texture as type 1 and pH as type 2 threshold variables.

*Estimated threshold parameters*^a^		

Type 2-pH	5.6^***^	
	(0.25)	

*Select regression results*	pH ≤ 5.6	pH > 5.6

Fertilizer rate	3.664^***^	3.30^+^
	(0.67)	(2.06)
Rate squared	−0.001^***^	−0.44 e^−3^
	(0.3e^−3^)	(0.7e^−3^)

*Clay group*		
1 – very low	*Subsumed*	*Subsumed*
	–	–
2	116.85	219.25
	(176.6)	(412.3)
3	333.72^*^	−262.57
	(187.6)	(371.7)
4 – more than about 40%	135.27	88.37
	(247.8)	(394.0)

Fertilizer rate × Clay group		
1 – very low	*Subsumed*	*Subsumed*
	–	–
2	1.528^**^	1.677
	(0.70)	(2.42)
3	1.074^+^	3.886^*^
	(0.72)	(2.26)
4 – more than about 40%	1.274^+^	2.966
	(0.90)	(2.61)
Weeks delay * Fertilizer rate	−0.364	−0.826^+^
	(0.25)	(0.56)
N	1002	451
R^2^	0.47	0.47
Weighted R^2^	0.47	

Robust standard errors in parentheses. *, **, *** Indicate significance at the 10%, 5% and 1% levels respectively, + indicates significance at the 15% level. Chow test rejects structural equality between Type-2 (pH) regimes (p = 0.00) a – thresholds standard errors are based on 50 bootstrapped replications.

Table 5a, Table 5b present mean AP estimates by texture and pH regimes similar to Table 3a, Table 3b for model 1. For a given pH regime and fertilizer type, these results are also consistent with *ex ante* expectations. The mean AP of fertilizers is lowest on the lowest clay content soils. Again, though, understanding the impacts of soil acidity on basal fertilizer requires more than a cursory examination of mean effects. Some results seem to suggest basal fertilizers are equally or more effective on more acidic soils, which is unexpected, but this result is partially explained by the importance of timely application. Here too, the estimated cost of delayed application is roughly twice as great on higher pH soils. The *potential* yield response to timely basal is more like the estimated effects of top dress, where response rates are the same or higher on high pH soils, especially when there is also a higher portion of clay particles.^14^ The estimated yield response to basal is low on high pH soils because *actual* applications are delayed.

**Table 5a:** Matrix of average product (kg/kg) of basal fertilizer for maize production in Zambia (Model 2).

		pH level	
		Low ≤ 5.6	High > 5.6
Soil texture group	Less clay − 1	2.02^***^ (0.66) [n = 84]	0.79 (2.41) [n = 33]
	2	3.53^***^ (0.73) [n = 205]	2.01^**^ (1.00) [n = 61]
	3	3.14^***^ (0.70) [n = 367]	4.33^***^ (1.17) [n = 145]
	More clay − 4	3.48^***^ (0.90) [n = 39]	3.45^*^ (1.97) [n = 23]

Source: RALS 2012. Delta-method standard errors in parentheses, sub-sample sizes in brackets. Note – APEs include fertilizer users only.

**Table 5b:** Matrix of average product (kg/kg) of top dressing fertilizer for maize production in Zambia (Model 2).

		pH level	
		Low ≤ 5.6	High > 5.6
Soil texture group	Less clay − 1	3.30^***^ (0.64) [n = 85]	3.12 (2.03) [n = 32]
	2	4.80^***^ (0.75) [n = 208]	4.73^***^ (1.56) [n = 64]
	3	4.34^***^ (0.81) [n = 376]	6.96^***^ (1.17) [n = 150]
	More clay − 4	4.53^***^ (0.90) [n = 40]	6.03^***^ (1.89) [n = 25]

Source: RALS 2012. Delta-method standard errors in parentheses, sub-sample sizes in brackets. Note – APEs include fertilizer users only.

### 6.3 Profitability and the robustness of findings to model specification

**Fig. 5:**
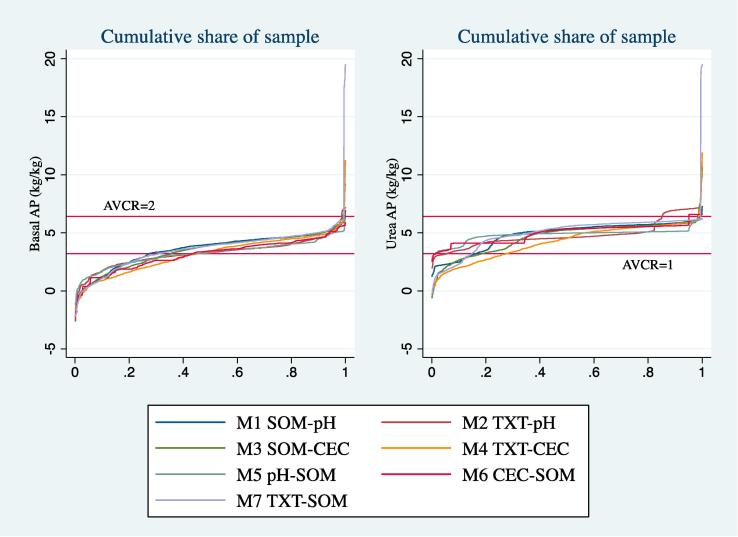
Cumulative distribution of average product of fertilizer estimates (amongst fertilizer users) under various model specifications. Notes: Model 1 (M1)-SOM = Type 1 (T1) threshold and pH = Type 2 (T2) threshold; M2-Clay = T1 and pH = T2; M3-SOM = T1 and CEC = T2; M4-Clay = T1 and CEC = T2; M5-pH = T1 and SOM = T2; M6-CEC = T1 and SOM = T2; M7-Clay = T1 and SOM = T2. Reference lines are at 3.2 and 6.4 marginal kg of maize per kg of fertilizer applied, which corresponds to average value cost ratios (AVCR) for 1 (fiscal profitability) and 2 (risk averse profitability as defined by Kelly (2002)) at prevailing prices for maize and fertilizer.

So far, we have only discussed results of two models out of several possible specifications. To show the robustness of our findings, Fig. 5 illustrates results from models 1 and 2 plus several additional specifications in the form of the cumulative distributions of estimated average product of basal and top dress fertilizer.^15^ Each of seven models (M1 to M7) are described in the legend and notes with the type 1 (T1) threshold variable listed first, followed by the type 2 (T2) threshold variable. M1 and M2 are “model 1” and “model 2” that were discussed above. Model 5 (M5), for example, is similar to M1 in that SOM and pH are the regime-defining variables, but in M5 pH is the type 1 variable and SOM is type 2 (i.e., the “types” for M1 and M5 are switched). Fig. 5 clearly illustrates our results describing the distribution of APs for the overall population are robust to these various model specifications.

Reference lines on Fig. 5 are at 3.2 and 6.4 kg/kg response rates. These respectively indicate average value cost ratios (AVCR) of 1 and 2 for fertilizer applications based on mean commercial prices paid for fertilizer (ZMK 3723/kg) and harvest time (May-July) commercial maize prices (ZMK 1177/kg) reported by farmers in the RALS12 data. An AVCR equal to 1 indicates the fiscal “break even” point, irrespective of transfer costs. In this case, the various models predict between roughly 25–45% of basal fertilizer users operate at a fiscal loss, as do up to 25% of top dress users. Kelly (2005) suggests an AVCR of at least 2 is required for smallholder farmers to adopt a technology to account for transfer costs and risk aversion. The texture and pH model (M2) suggests about 15% of farmers can expect such returns. Otherwise, using Kelly’s (2005) criterion we would conclude virtually no farmers have sufficient incentive to use fertilizer regardless of fertilizer type or model specification. For the majority of farmers, the estimated response rate and prevailing prices combine to form an average value cost ratio somewhere between 1 and 2, meaning if the outcome had been known a priori, and excluding transfer costs, fertilizer use would have been a fiscally rational decision. Since outcomes are uncertain, however, lacking a priori knowledge on precisely how yields would respond to fertilizers would give most risk averse farmers sensible pause before deciding whether to adopt fertilizer or not.

## 7 Conclusion

In this article we incorporate farm management and household data with soil analysis to understand and quantify the variable and low response rates to and profitability of inorganic fertilizer applications in Zambia. Unlike earlier work, we develop models that simultaneously allow for the effects of multiple soil characteristics and critical thresholds. We estimate threshold effects on yield response to fertilizer between pH levels of 5.4 and 5.6, soil organic matter levels 1.2–1.4% and we find significant texture-related effects. Alternative models also incorporate these characteristics with cation exchange capacity to demonstrate robustness to model specification. Several average yield response estimates range from not significant to 7 maize kg/fertilizer kg, depending on soil characteristics. We find fertilizer use unprofitable at commercial prices for many Zambian fields under current farm management practices. Even if transfer costs are ignored, for example, up to 45% of fertilized fields have an estimated average value-cost ratio for basal fertilizer less than one at commercial prices.

Several implications emerge from this study. First, it is well known that soil characteristics vary widely across Africa and even within countries. Nevertheless, far more policy focus is given to encouraging the use of inputs than to the appropriateness of the technologies being promoted. The recommended application rates for fertilizer in Zambia are constant, despite a landscape that is clearly heterogeneous in important and relevant ways. From our own interactions with the leaders of the Zambian extension agencies we know part of the reason fine-tuned recommendations do not exist is due to lack of policy priorities, which in turn has led to little change in farmer practices over the past decades.

Our article contributes to a growing literature that consistently suggests agricultural input effects on yield can be dramatically limited or enhanced by prevailing soil conditions. We believe it is important to continue pursuing this vein of research so as to build knowledge regarding the actual effectiveness of the inputs being used and promoted, the determinants of that effectiveness on farms (as opposed to field trials) and to identify possible complementary or substitutive practices that could sustainably increase farm yields.

In some cases, affecting change in prevailing soil characteristics is not a realistic policy goal. Soil texture (the percentage of sand, silt, clay), for example, must be considered constant under feasible agricultural practices for Zambian farmers. Coarse textures, which our results suggest limit yield response to fertilizer, can change if large amounts of clay are brought in, but this is rarely done and would be difficult. On the other hand, different agricultural management can affect soil structure on a given texture, which is the assemblage of particles and organic matter in aggregates of different sizes. So, while policy may not expect to change soil texture, there are large potential benefits to developing policies that increase the understanding of farmers and extension agents with respect to best practices on given soil textures.

In other cases, soil characteristics can be changed. It is known, for example, that adding lime will increase pH on acidic soils (in addition to adding some nutrients), but this is often assumed to be cost prohibitive (Burke et al., 2017). Funding research into other ways to raise pH or alternative ways to apply lime may find beneficial ways to improve soil pH or discredit the assumption about the fiscal feasibility of effective liming.

The second implication of our findings is that promoting timely fertilizer application could be a potentially powerful lever to increase the productivity of blended fertilizers. Farmers often quite sensibly delay applications to avoid risk of wasting applications on seeds that do not germinate. Another cause for delay, at least in Zambia, is frequently a lack of availability because government acquired fertilizers are delayed due to payment or customs issues. Addressing farmer risk aversion is potentially a very powerful policy lever, but a difficult one to pull. Avoiding delays due to administrative issues is more likely to be a tenable option.

Third, if our findings hold and fertilizer cannot be used profitably in many rural areas, manipulating consumer prices to ensure availability of fertilizer may represent a suboptimal use of resources. If the additional revenue from fertilizer cannot cover the full economic cost of delivering it to farmers, then fertilizer subsidy programs, for example, may represent an unsustainable way to promote fertilizer use. Increased fertilizer use could be better promoted through extension and adaptive research that enables farmers to raise the profitability of using fertilizer through managing their soils more effectively and sustainably.

Research and extension are policy levers that appear to be underutilized. By nearly all accounts, developing and sharing knowledge regarding efficient input use will more effectively lead towards sustainable intensification than the naïve and pan-national recommendations for fertilizer types and application rates. In Zambia and other African countries, agricultural research institutions are underfunded and extension agencies are essentially defunct, largely because the bulk of financial and human resources are dedicated to subsidy programs (Jayne and Rashid, 2013, Jayne et al., 2018). Diversification of productivity enhancing strategies is another potentially very powerful policy lever.

One might view our findings as discouraging. On the other hand, one might consider the array of under-promoted and under-employed alternatives to naïve fertilizer recommendations as readily available opportunities to dramatically affect yields in the near and long-term. Producing more on the same land, with the same or fewer inputs, increasing rural farm incomes and the advantageous social outcomes that go with it are attainable goals. Accomplishing these goals, however, is almost certainly going to require the scope of prevailing strategies to broaden. The sooner the better.

## Footnotes

1 Maize is by far the most commonly grown and consumed staple food in Zambia

2 The macronutrients not discussed in the text are calcium, magnesium and sulfur; the micronutrients are boron, chlorine, copper, iron, manganese, molybdenum, nickel and zinc.

3 “Fertilizer” means water-soluble inorganic fertilizers. We use “element” and “nutrient” interchangeably.

4 CEC is measured in milliequivalents (meq) per 100 g of soil.

5 Related measurements are organic carbon content or soil carbon content. These measures are highly correlated, and can effectively be thought of as rebased measures of each other.

6 Weeding was quantified simply as “number of weedings”. A more detailed enumeration of this management practice would improve future analyses.

7 Moving forward, variable names such as “*yield*” are in italics.

8 The full questionnaire is available on-line at http://fsg.afre.msu.edu/zambia/2012_Rural_Agricultural_Livelihoods_Survey%28RALS%29.pdf.

9 The smaller questionnaire has not been posted on-line, but can be made available.

10 If one were to examine Fig. 1, we argue there are not many other sensible ways to group these categories. Here again, part of our motivation is data limitation - with more data it would be preferable to allow each soil texture category stand alone.

11 It is important to note George et al. (2012) do not specify whether they measured pH in Cacl2 (as we have) or in water. This would affect the comparability of results.

12 We also control for omitted farmer ability with our education proxy, though admittedly the correlation between this proxy and the omitted variable it represents is probably less strong than that between soil characteristics and otherwise ambiguously defined “soil quality” (which is why we treat education as a “control” variable and not a “key” variable subject to scrutinous interpretation).

13 The same can be said about omitted variable bias that may remain despite our proxy for farmer ability.

14 In an appendix available upon request, a figure shows the expected APs for top dressing across pH values in a way similar to Fig. 3 for several of the different robustness check specifications.

15 Full results are available upon request.
